# In-vivo design feedback and perceived utility of a genetically-informed smoking risk tool among current smokers in the community

**DOI:** 10.1186/s12920-021-00976-1

**Published:** 2021-05-26

**Authors:** Jessica L. Bourdon, Amelia Dorsey, Maia Zalik, Amanda Pietka, Patricia Salyer, Michael J. Bray, Laura J. Bierut, Alex T. Ramsey

**Affiliations:** 1 Wellbridge Center for Addiction Treatment and Research, Center for Addiction Science, 525 Jan Way, Room 1523, Calverton, NY 11922 USA; 2 Department of Psychiatry, Washington University in St. Louis, St. Louis, MO USA

**Keywords:** Smoking, Genetics, Cessation, Implementation

## Abstract

**Background:**

The use of genetically-informed personalized risk information for behavioral disorders, namely smoking and smoking-related behaviors, is a promising yet understudied area. The Genetics and Smoking Risk Profile, or *RiskProfile*, leverages genetic and environmental information to communicate one’s risk for smoking-related diseases. Although prior studies have examined attitudes toward genetic results, little research has investigated these perceptions through a lens of in-vivo testing; that is, user-centered design feedback in response to personalized genetic results being returned contemporaneously. This qualitative study engaged current smokers in usability testing of the *RiskProfile* within the context of concurrently receiving this personalized, genetically-informed smoking cessation intervention.

**Methods:**

Eighty-nine participants who were current smokers responded to open-ended interview questions on perceptions of smoking-related genetic information and the content and format of the *RiskProfile* intervention that they had received moments before. Data were analyzed via the conventional content analysis approach in which themes were allowed to emerge throughout the analysis.

**Results:**

Participants were able to reference and offer design input on specific elements of the *RiskProfile*. Overall, current smokers perceived the *RiskProfile* to have high potential utility. Constructive feedback that current smokers offered about the tool centered around suggested improvements to optimize its usability and technical content.

**Conclusions:**

The detailed and constructive feedback from participants highlights that in-vivo feedback offers a useful design approach that addresses concerns of rigor and relevance when returning genetic results. This unique method demonstrated perceived utility and constructive design feedback for the *RiskProfile* among current smokers and can play an important role in optimizing the design and implementation of personalized genetic risk interventions moving forward.

**Supplementary Information:**

The online version contains supplementary material available at 10.1186/s12920-021-00976-1.

## Background

Despite numerous strides in the precision medicine initiative [1], there remains a lack of widespread use of genetic information in clinical and community settings among individuals with behavioral health disorders such as smoking [2]. Pharmacogenomic advances continue to enhance treatment for breast cancer [3], psychiatric conditions [4], and dosing for specific drugs such as the anticoagulant warfarin [5, 6]. Yet, relatively less attention has been paid to the prospect of utilizing genetic information to communicate personalized risk and promote health behavior changes, such as use of evidence-based pharmacotherapy and quitting or reducing smoking [2] [e.g., 7–9, 10–12]. Genetic information has the potential to guide prevention and treatment efforts across disorders [9, 13], especially when considered in tandem with environmental or lifestyle information [14].

There is noted public interest in genetic testing for psychiatric disorders, including disorders of addiction. That interest varies based on context (e.g., having symptoms of a disorder, having a family member with a diagnosis, age, entertainment value, skepticism) and perceived potential impact and value of the genetic results [15, 16–18]. To meet this demand, numerous studies have assessed stakeholders’ perceptions about the hypothetical use of genetic information in healthcare [19–26, 27] as well as the consequences of returning actual genetic information to patients and consumers [10, 12–17, 27–31]. This work spans multiple settings, designs, types of genetic information (e.g., direct-to-consumer testing, medical genomic sequencing panel results), and ways of presenting genetic information (e.g., active versus passive). Collectively, this work has noted many barriers and facilitators toward implementing genetic information into healthcare. Pressing barriers include disagreement over who is responsible for discussing genomic information with patients and consumers and, relatedly, the insufficient supply of genetic counselors to meet the growing demand for personal access to genomic information [8, 22, 24, 32–36, 37]. There are also concerns over inadequate opportunities for education and training about genetics as a whole [22, 25, 26, 34] as well as fears of insurance discrimination [19, 15]. Implementation facilitators include the relative lack of evidence for adverse patient and consumer reactions to receiving genetic information [7, 16, 30]. Gains in knowledge, self-efficacy, and overall engagement with their results have also been noted among patients and consumers [28–30] in addition to positive behavior changes such as disclosing results with another person or lifestyle changes [12, 17, 31, 38].

To facilitate optimal implementation of genomics in behavioral health, a working group was recently established to continue bridging this gap between genetic information and behavioral health [39, 40]. There is now a growing body of work on the impact of genetic information about smoking and smoking-related disorders (e.g., nicotine addiction, lung cancer). These studies have informed current smokers’ perceptions about hypothetical and actual genetic results [41, 42, 43] and have assessed behavior change, decision making, motivation, and other related outcomes subsequent to return of personalized genetic results [7, 44–46, 11, 12, 17, 18, 47]. Despite these studies providing a wealth of important findings, no known studies have solicited user-centered design feedback of a genetic risk communication tool concurrent with the return of smoking-related genetic results. That is, studies have not examined the “in-vivo” perceptions of current smokers when they are, in the moment, engaging with personalized genetically-informed interventions. As such, there remains a scientific gap involving user-centered design feedback on personalized genetic risk tools using methods that maximize personal and contextual relevance and minimize recall bias. To maximize the rigor and relevance of this evidence, user-centered research methods may help researchers and clinicians gain a better understanding of an innovation as individuals concurrently engage with the innovation.

A key scientific gap is on perspectives from current smokers in the context of receiving personalized genetic information. Methods from the fields of implementation science and design thinking can aid in establishing the consistent use of genetic information for behavioral disorders in real-world, contextually-relevant scenarios by uncovering innovation-, individual-, organization-, and system-level barriers to integration and use of this evidence [48, 49]. An important next step that will build upon past research is to ensure that the tools used to communicate genetic information for behavioral health are designed in a way that respond to known implementation barriers and facilitate the use of genetic information to positively impact patient empowerment, self-efficacy, and behavior change [50, 51].

### Study aims

The aim of the current study was to qualitatively analyze participants’ “in vivo” perceptions and feedback on a genetically-informed smoking risk tool within the context of concurrently receiving this personalized tool as part of a proof-of-concept intervention study [52]. Following genetic testing via 23andMe, current smokers received a personalized, genetically-informed smoking cessation intervention. The intervention included the smoking risk tool, hereafter referred to as the Genetics and Smoking Risk Profile, or *RiskProfile*. The *RiskProfile* was previously developed and initially validated [53] and then subsequently demonstrated utility in motivating progress toward smoking cessation, including significant pre-post reductions in cigarette smoking [52]. The current study applied in-vivo methods to qualitatively investigate and further inform the design of the *RiskProfile*, an approach that builds upon prior return of smoking-related genetic results studies and may be easily incorporated into future studies.

## Methods

### Genetics and smoking risk profile (*RiskProfile*)

The *RiskProfile* was created in an iterative manner whereby its design was optimized using separate samples of participants (see 53 for more details). It consisted of a tri-fold brochure that first presented a visually-appealing introductory outer panel that primed participants to expect information about their genetics and smoking-related outcomes (see Additional file 1: Fig. 1: How genes and smoking impact my risk; reproducced with permission from 35). The inner flap provided an overview of the 23 human chromosomes with chromosome 15 highlighted as it was specifically examined in the study. The larger inner panel provided personalized results that revealed the genetic variants for specific single nucleotide polymorphisms. These genetic results were combined with phenotypic information (i.e., number of cigarettes per day at baseline) into an algorithm that determined the individual’s risk for developing each of the three smoking-related disorders (lung cancer, lung diseases such as chronic obstructive pulmonary disease (COPD), and difficulty quitting smoking) (see https://osf.io/tmwyn/ for an overview of the algorithm). The large, inner panel also provided actionable information about the benefits of healthy behavior changes. The back panel referred the individual to resources to help with smoking cessation and included Quitline-, text-, and app-based tools, as well as potential medications to discuss with their physician. See Additional file 1: Overview of the *RiskProfile*'s contents.

**Table 1:** Overview of feedback on the RiskProfile

Domain	Theme	Meaning	Quote
Motivations for receiving the *RiskProfile*	Strong interest in the genetics of smoking	Broad interest in the genetic underpinnings of smoking and personal interest in individualized risk for developing smoking-related disorders	“I guess how [smoking] related to my DNA, and if there was something [the *RiskProfile*] could tell me that I didn’t already know.”—*Male, aged 60–69*
	Information-seeking	Wanted to gain specific information about smoking, such as how to quit, risk for smoking-related disorders (not a genetics-related interest), and why they smoke	“I really wanted to know is it me being stupid and stubborn or is there something more to why it’s been so hard for me over all these years?”—*Male, aged 40–49*
Perceived utility of the *RiskProfile*	Informing and educating	Found that the tool helped provide useful information about the risks of smoking as well as education about their own health and risk	“It’s just helpful by putting it in your face, honestly. […] I know a lot of people, including myself, who can be in denial about how at risk you are and [the *RiskProfile*] puts it out there in front of you.”—*Male, aged 20–29*
	Motivating	Noted that the tool was motivating for smoking cessation attempts and lifestyle changes	“I think it could help people realize there is a reason that they’re craving cigarettes or nicotine. And have them be a little more serious about looking for […] extra added aids and quitting. Instead of just trying to […] quit cold turkey.—*Female, aged 40–49*
Potential concerns about using the *Risk Profile*	No concerns	Noted that they did not have any concerns about using the tool	“Do I have any concerns about it? No, I wholeheartedly agree with it.”—*Male, aged 70–79*
	Lack of utilization	Potential for the tool to be under-utilized or not utilized at all for a variety of reasons	“Getting the information and not following through with […] trying to stop”—*Female, aged 60–69*
	Privacy	Fear of genetic information being misused by third parties, including 23andMe, insurance companies, and the government	“My only fear is that [my genetic information] may get out to the public and insurance companies may use that against me.”—*Male, aged 40–49*
Suggestions for improving the effectiveness of the *RiskProfile*	Leave as-is	Noted a preference for not revising the tool	“No, I don’t know. I can’t think of anything.”—*Male, aged 40–49*
	Improve jargon, technical details, and layout	Noted ways that the formatting and content of the tool could be enhanced	“Dumbing it down a little bit so that you’re not talking about […] specific chromosomes, and gene markers, and stuff. I can’t follow that.”—*Male, aged 20–29*
	Improve impact and meaningfulness	Centered around more personalized improvements that could be made to the tool	“Well if there was a way to pinpoint which smoking cessation aid would be best for a specific person.”—*Female, aged 60–69*
General positive feedback	–	Offered in all domains about various aspects of the tool	“I think everything I’ve seen so far is fantastic.”—*Male, aged 20–29*

Discussion of the *RiskProfile* with participants was scripted and (1) gave an overview of genetics in lay terms, (2) emphasized the importance of genes and environment in smoking-related behaviors and diseases, (3) encouraged cessation, or reduction of smoking if full cessation was not possible, and (4) acknowledged the early stage in which this research was being conducted, noting that new genetic markers for smoking-related diseases are still being discovered and that feedback on the *RiskProfile* would be valuable for improving future research and practice.

### Study protocol

#### Study context

As part of the genetically-informed smoking cessation intervention, participants had been recruited into a three-visit feasibility study to demonstrate proof of concept for the *RiskProfile* [52]. Participants were recruited into the study using a variety of both active and passive methods, including existing institutional registries, posted flyers, and online ads (see 53 for more details). Participants aged 21 or older who were current smokers of tobacco from the [greater St. Louis, MO] region were eligible for the study. In Visit 1, participants provided informed consent, answered baseline questions about their current smoking and perceptions of using genetic results, and provided DNA via saliva sample to be sent to 23andMe. Participants consented to being interviewed and audio recorded during visits, giving their saliva as part of the 23andMe process, and allowing study staff to analyze their raw genetic data to generate the *RiskProfile*. The consent form also included information about how participants’ biological samples would be treated, as well as privacy, risks, and benefits of the study (full informed consent form is available at https://osf.io/tmwyn/). The research team utilized the raw data from the 23andMe genetic reports and self-reported ancestry information to create the *RiskProfile* (see above). Specifically, we selected clinically valid genetic markers in the *CHRNA5* gene region that have been identified in multiple genome-wide association study as having robust associations with nicotine dependence, smoking-related lung cancer and other lung diseases, and difficulty quitting smoking. Participants were then invited to Visit 2 to assess their current smoking and to receive and discuss the results of their *RiskProfile.* Participants also provided feedback about their “in-vivo” reactions to the tool and its potential utility for promoting smoking cessation; this served as the basis for the current study. Importantly for Visit 2, because all participants were current smokers, the *RiskProfile* by design never communicated a message of “low risk”, despite the participants’ genetic risk results. Further, all participants were given a consistent, strong recommendation to quit smoking and referral to freely accessible smoking cessation resources. Visit 3 was a phone-based, brief follow-up assessment to assess potential change in current smoking one month following the intervention. Analyses for that visit are outside the scope of the current qualitative study (see 52).

#### Current study

The present study was conducted as part of Visit 2 in the three-visit intervention study. Using a standardized verbal script (see https://osf.io/tmwyn/), a trained research team member presented each component of the *RiskProfile* (e.g., the concept of genetics, their risk) to participants, provided guidance on how to interpret the results, and offered multiple opportunities to ask questions. After the tool was delivered, the presenter left the room and a trained interviewer asked semi-structured, open-ended interview questions about the *RiskProfile* tool: (1) *When you first signed up for this study, what did you most want to learn in regard to your smoking behaviors?* (2) *How might a tool like this genetics and smoking risk profile be helpful in guiding smoking cessation attempts?* (3) *What are some of your concerns with using a tool like this profile to guide smoking cessation attempts?* (4) *How could this genetics and smoking risk profile be improved to help motivate smoking cessation attempts?* Participants were also asked to share any final thoughts about the content or format of the *RiskProfile*, and interviewers were trained to prompt participants as necessary throughout the interview. Participants were monetarily compensated $25 for this visit.

### Analyses

Transcription of in-vivo responses followed a verbatim approach whereby responses were written exactly as they were said by participants except that filler words (e.g., “um,” “like,”) were filtered out to aid in analysis [54]. A co-author (AD) on the paper conducted the transcriptions. Qualitative analysis followed a conventional content analysis approach where themes were allowed to emerge from the data [55]. The order for analysis included creating open codes, collapsing codes into categories, and then conceptually ordering the categories into themes in an inductive manner (see Additional file 1: Table 2: Breakdown of initial codes, categories, and themes across questions). Microsoft Excel was used to analyze and count the codes, categories, and themes [56, 57], and multiple codes per response were allowed (see Additional file 1: Text 1: Details about coding with Microsoft Excel). Researchers engaged in constant comparison whereby codes and categories were checked throughout data analysis to ensure their continued relevance and appropriateness. This is necessary because later responses can inform earlier codes and vice-versa [54]. Relevant quotes were identified as necessary.

Each question was analyzed by two raters (JLB and AD) who regularly met to discuss similarities and differences in their analyses. Analyses often aligned except for specific codes used (e.g., semantic differences) or the structuring of themes (e.g., one person may have summarized positive and negative feedback as one theme and the other may have divided it into two themes). All coding discrepancies were discussed and resolved collaboratively by both raters to achieve consensus for each question before continuing the analysis.

## Results

Results are presented as five domains; relevant themes accompany each one and all are summarized in Table 1. The domains were structured around the original questions asked to participants, although responses from multiple questions comprise each domain.

### Sample demographics

The sample consisted of 89 participants (mean age = 47 years; female = 51%; White/Caucasian = 56%; Black/African American = 36%; another ancestry = 8%; Hispanic or Latinx = 2%). All participants consented to being audio recorded for transcription except for two. Summaries of those participants’ responses were recorded via written record.

### Domain 1: motivations for receiving the *RiskProfile*

Motivations for receiving the *RiskProfile* centered upon two different themes of smoking—strong interest in the genetics of smoking and information-seeking for broader health reasons.

#### Strong interest in the genetics of smoking

Most participants were motivated by their interest in the genetic component of the study, and some were engaged on a personal level in which they deeply reflected and drew substantial meaning from the information being presented to them. For example, one participant wanted to know “if I had any genetic factors involved [in smoking-related outcomes]” while another simply wanted to know if there was “a genetic variant that caused you to be more addicted to the tobacco or … made it harder to quit.”

Interestingly, even participants who were not well-versed in the genetics of smoking were often familiar with the influential role that genetics plays in smoking and related behaviors. For example, one participant noted “I wanted to […] just see more about how the addictive gene affects our family and me personally.” Others discussed genetics in a deterministic manner, implying that they either thought or hoped that it explained their smoking behaviors more than their environment (e.g., lifestyle or behavioral factors). As one participant stated, “So I guess I was interested in the genetic aspect of it to see if there were factors working against me that I may not have a lot of control over.”

#### Information-seeking for broader health

There was a subset of participants who participated in the study to gain very specific types of information about their health. For example, some participants were interested in their overall risks for disease and smoking-related illnesses and how this could impact their health. Some participants even hoped for a formal diagnosis or prognosis from this study. Others wanted information to support their efforts to quit smoking (e.g., cessation medications). As one participant noted, “I wanted to learn how hard it would be to quit and what would be the best way to quit.”

### Domain 2: perceived utility of the *RiskProfile*

Participants focused on two key themes of the *RiskProfile*’s utility—how it can inform and educate or potentially motivate behavior change.

#### Informing and educating

Participants commented on how useful it is for individuals who smoke to know their genetic risk because that can inform future health and behavior decisions. As one participant said, “I wasn’t sure how high of a risk I was [for smoking-related outcomes], but to see I was in the higher risk category—that was pretty eye opening. And if I ever have children too, it would be good to have some kind of knowledge to help make an environment that they wouldn’t want to smoke [in].” The *RiskProfile* can also help inform individuals about why they smoke and/or struggle with craving nicotine. Although most participants were already aware of the harms of smoking, several were of the mindset that more information and education, particularly when presented in a unique and more personalized way, was useful. One participant stated, “I think it’s just one more bullet in the chamber, one more weapon in your belt, however you want to put it.”

#### Motivating behavior change

Several participants commented on the various ways that the *RiskProfile* can help participants focus on their health and the long-term goal of quitting because of its motivational impact. For example, one participant stated that the tool was “Just more motivation. It tells you how critical it is to […] attempt to quit.” As one participant noted, “I think […] it will help [others] change their lifestyle and I would like to think that it’s going to help me change my lifestyle.”

### Domain 3: potential concerns about using the RiskProfile

Participants expressed a range of potential concerns about using the *RiskProfile*. These included having no concerns, concerns about under-utilizing the tool, and privacy concerns.

#### No concerns

It is worth noting that the majority of participants did not have any concerns about the genetic smoking risk tool. Participants generally remarked on how they did not find anything concerning with the smoking risk profile tool and mentioned that it could be helpful to use. For example, one participant commented “No, no concerns. I love the idea.”

#### Lack of utilization

Many participants were concerned about the *RiskProfile* being under-utilized by themselves and others moving forward. Some of these concerns involved personal agency following receipt of the tool, for example, including acknowledgment of now facing the difficulty of cigarette reduction or cessation and fear of such efforts failing. A couple of participants also noted the concern that the tool might not be used after the visit, possibly because it is simply not useful enough for individuals who already know the risks of smoking. For example, one participated noted, “I know the hazards [of smoking] and […] at a certain point […] we just don’t care because a lot of [us] figure, ‘I’ve lived my time.’” Additional concerns were about potential unanticipated consequences of the *RiskProfile* and whether the tool could be enabling for individuals with a low genetic risk for smoking-related outcomes. As one participant said, “I don’t so much have any concerns besides [someone] being at genetically lower risk and thinking that they have a free pass to smoke now because they’re at low risk.”

#### Privacy

Many participants expressed concerns about the privacy of their information, but not about the information in the *RiskProfile* itself. These concerns were mitigated by participants’ trust that the research team associated with this project were upholding the highest possible privacy standards, but such standards were not assumed to be upheld by third parties. Participants expressed broad concerns about the safety of their genetic information, third party tracking, distribution, and use of their genetic information (e.g., insurance companies).

### Domain 4: suggestions for improving the effectiveness of the *RiskProfile*

Participants provided a range of feedback about the *RiskProfile*, including leaving it as-is, improving the tool’s content, and improving the tool’s impact.

#### Leave it as-is

Although several participants noted areas of improvement for the *RiskProfile*, most had no suggestions and/or voiced approval. As one participant said, “I think that it did what it was designed to do. I don’t know how it could be improved.”

#### Improve jargon, technical details, and layout

Some participants commented on the formatting, layout, information, and content of the *RiskProfile*. Although these comments were quite diverse, they did provide concrete suggestions to improve the tool in order to better motivate cessation attempts. Feedback on key areas of improvement included simplifying the layout, adding more information about the genetic information that went into making the profile risk score, and improving the language because the tool was not intuitive and required explanation. Some participants also suggested to include risk for family members. Finally, participants recommended periodic updates to the tool as new information (e.g., relevant genetic markers, risk percentages, cessation aids) are made available.

#### Improve impact and meaningfulness

There were several responses that provided insight beyond the format or content of the tool itself. Many participants offered improvements that would help make the *RiskProfile* more impactful and meaningful, thus potentially improving motivation to attempt smoking cessation. Some suggestions included expanding what went into the risk profile score, such as asking more information about behaviors, environment, markers, and family history. Other suggestions included ways to make the experience more personalized, such as offering information about personalized smoking cessation. Other suggestions of how to maximize the impact and meaning included increasing the perceived severity of disease risk such as more sensationally displaying the potential consequences of continued smoking (e.g., that individuals with COPD often use oxygen tanks) and arranging follow-up professional outreach with a healthcare professional. A few participants also discussed ways to improve the reach of the study, such as more advertisement and targeting younger adults. This feedback highlights the importance of reaching and engaging the right audience with highly personalized and actionable content, all of which is perceived to be essential to more widespread implementation of this tool.

### Domain 5: general positive feedback

There was much generalized positive feedback offered by participants that cut across all of the other four domains. Participants seemed happy about the experience as a whole, from the in-person aspect of the study to the personally engaging information that the *RiskProfile* provided. Many noted that the tool was helpful and motivating and that the information presented was unavoidable and eye-opening. Additional positive feedback about the *RiskProfile* centered around the fact that the information was relevant and credible, the tool itself would be useful for young people (e.g., prevention), and the layout was appropriate. Notably, one participant specifically liked that even though it was a personalized tool, there was no identifying information on the tool itself. As they said, “I mean you’re handing me a paper that doesn’t have my name on it so even if I dropped it, nobody’s going to know it was me.”

## Discussion

The current study qualitatively analyzed in-vivo responses about a personalized, genetically-informed Genetics and Smoking Risk Profile (*RiskProfile*). As a whole, participants expressed favorable views of the *RiskProfile*, providing detailed information about specific components of the tool that would not have been available without the in-vivo design. Results across five inter-connected domains provide a richer understanding of current smokers’ motivations to participate, the specific perceived benefits and potential utility of the *RiskProfile*, and the concerns and suggested opportunities to improve this tool. To date, few studies have studied how to translate responses to personalized genetic susceptibility information into evidence-based interventions designed to communicate disease risk and motivate behavior change [15, 58, 59]. Thus, the current study expands past research in at least two ways: the use an in-vivo study design to concurrently study the genetically-informed tool as it is being delivered to participants; and the ability to apply this rich, highly-specified in-vivo design feedback to improve the content and format of the *RiskProfile* moving forward.

### Relative advantage of the concurrent in-vivo design

The in-vivo approach used in the current study extends prior research that examined participants’ attitudes about receiving and using both hypothetical [19, 23, 41, 42, 60, 27, 47] and personalized [7, 10–18, 28–38, 47, 2] genetic information for various purposes by analyzing participants’ feedback about the *RiskProfile* concurrently with its delivery. This study capitalized on the opportunity to engage participants using qualitative and user-centered design methods to gain a deep understanding of their motivations, perceived utility, concerns, recommended improvements, and positive aspects of the *RiskProfile*. This was done from the context in which participants received the personalized genetic risk tool. This approach yielded detailed, contextually-relevant, and timely feedback. Thus, such deep, in-vivo analyses appear advantageous in gaining an understanding of the potential utility of personalized genetically-informed tools. Accordingly, the information provided by participants will influence future iterations of this work in ways that would not have been possible without the current design. Future studies that utilize personalized genetic information may be able to optimize the design of genetically-informed tools by implementing a similar methodology.

Although current smokers will likely benefit from behavioral interventions that are more closely aligned with their participatory design input, this in-vivo method carries important advantages for researchers as well. For instance, this type of in-depth, contextually-relevant qualitative feedback design may be particularly sensitive to detecting plausible mechanisms of behavior change that warrant additional investigation in future research. Potential behavior change mechanisms that were discussed by participants across domains included health-related cognitions (e.g., perceived risk of disease, perceived benefits of smoking cessation, perceived value of cessation aids) and engagement factors (e.g., personal relevance, comprehension, sharing of results) [61, 62]. To date, most studies that communicated genetic susceptibility information to participants have had little impact on behavior change, including smoking cessation [45, 12, 17, 28, 63]. However, there are notable exceptions [46, 52] as well as a more recent meta-analysis to suggest that genetic risk feedback may motivate behavior change among at-risk individuals [31]. These findings highlight the need for additional research focused on the potential mechanistic effects of genetically-informed behavioral interventions and the thoughtful design of these interventions to activate identified behavioral targets.

### Ability to quickly apply in-vivo results

Due to the current study’s design, there are three key take-aways that were only made apparent due to the in-vivo design of the study and which can immediately be applied to next steps in this research trajectory (see *Challenges and Future Studies* below). While these take-aways are specific to the *RiskProfile*, they offer insights for other teams who are currently working on returning genetically-informed information to participants. First, the overarching positive feedback demonstrated broad support among current smokers for the *RiskProfile*, a novel genetically- and environmentally-informed risk score that cannot be obtained through any direct to consumer or other third-party genetic testing services. However, the information provided by the *RiskProfile* may not be sufficiently motivating for all levels of smokers. Reflecting upon some participants’ responses about prevention opportunities and “targeting youth,” it is possible that this risk profile would have value for prevention programs or early smoking cessation efforts among new smokers. For the tool to be maximally beneficial among adult (and presumably longer-term) smokers, concerns raised in the current study need to be addressed. These include expanding the parameters of the risk profile (i.e., genetic and environmental information), addressing privacy concerns, consulting with a smoking cessation expert and genetic counselor, and receiving feedback about participants’ experiences with the *RiskProfile* after more time has passed and they can reflect more deeply about the tool.

Second, the genetic and environmental components that comprised the risk score in the *RiskProfile* need to be expanded. This addresses feedback from across all five domains. Many participants wanted specific information about risk to family members, education about risk to self and family, enhanced post-visit utility, and improved meaningfulness of the tool. In the future, the risk score algorithm and tailored intervention components could be supplemented with information about family history of smoking, smoking triggers, household and partner smoking, and other related factors. Including a more well-rounded risk score may make the tool more motivating and comprehensible to participants. It may also naturally create more dialogue between the researchers and participants.

Finally, there is a tension between the intensity and amount of information in the *RiskProfile*, and this relates to the modality in which the information is delivered. For instance, participants who thought that the tool did have enough information may be assuming that there will always be a researcher to explain the report in real-time. Conversely, those who think it has enough or too much information may be assuming the report will ultimately be a “stand alone” tool that needs to be very concise and easy to grasp without additional support or explanation from health professionals. Balancing this tension will be critical as the *RiskProfile* is expanded and updated in the future.

### Challenges and future studies

There will be challenges moving this research forward. A key one will be overcoming concerns with genetic testing, including ownership and use of the data [19, 15, 64]. These concerns were echoed via privacy concerns in the current study, although they were tempered by the trust in the research team, which may generalize more broadly to healthcare professionals. Also, as documented in the larger proof of concept study [52], it is encouraging that participants ultimately expressed no decision regret in association with their participation by the end of the study. There is also the barrier of genetic literacy and expectations about what the *RiskProfile*, or future iterations of it, can offer to participants. Despite careful wording and the use of scripts during the visit, some participants still expected to learn information that went beyond the scope of this study. Setting clear and reasonable expectations of participant benefit, while also acknowledging the range of perceptions of potential benefit, reflect another important tension to balance in future in-vivo research [64]. Relatedly, reinforcing the educational component of the tool will clarify participants’ expectations, as education is an oft-cited barrier to the translation (e.g., implementation, uptake) of genetic information among multiple stakeholder groups [21, 26, 34, 64, 65].

There are three main layers of examination and validation that will drive future directions of this research. First, immediate improvements can be made to the tool based on the current in-vivo feedback, and acceptability and feasibility should be iteratively assessed via in-vivo feedback with qualitative and quantitative metrics in future versions of the *RiskProfile*. Second, efficacy and effectiveness testing are needed to demonstrate clinical utility of the *RiskProfile* as measured by the tool’s impact on smoking cessation and other risk-reducing behaviors. Results from this study also suggest the need for dedicated testing of mechanisms, including cognitive (e.g., perceived risks and benefits, perceived value of cessation aids) and engagement-related (e.g., personal relevance) factors, that may uncover essential intervention targets for future research. Finally, as the focus of this study was on individuals in the community who smoke, it is unknown to what extent this type of tool would or should impact the smoking cessation care that healthcare professionals provide. It is plausible that patient-specific genetic and clinical risk information could facilitate patient-centered discussions and highlight the urgency and potential benefits of smoking cessation for healthcare professionals; however, to change their clinical practice, healthcare professionals must be convinced that this tool provides unique and useful insight. If found to be clinically useful, a future step would be to integrate the *RiskProfile* into community health agencies or clinical settings with healthcare practitioners delivering the tool to participants. Alternatively, an online version of the *RiskProfile* could be implemented in the future, such that individuals could access their personalized results and interpretation of those results, without the need to attend in-person counseling.

## Limitations

This study is not without limitations. First, as a qualitative study, we prioritized saliency of expressed responses to construct thematic content rather than quantitative metrics that often provide more summative evaluations. Relatedly, although we encouraged honest critiques of the usability and usefulness of the tool, it is possible that participants were reluctant to share more harshly negative feedback. Second, the potential confounding effects of the study staff were not examined (i.e., effects of who explained the genetic information, who administered the questionnaires during the visit, who coded the data). Third, it should be noted that organizational and clinical guidelines do not yet support the return of genomic information to participants for clinical reasons. Therefore, the current study was not designed to inform clinical decisions and participants were encouraged to speak with their physician about smoking cessation efforts. For instance, given the large consumer interest in reduced risk nicotine options, such as nicotine vaping products, some participants may have desired clinical recommendations about these alternatives with respect to their specific genetically-informed risks. Although we were unable to include such recommendations in this study, examining the utility of lower risk nicotine products in relation to genetic risk, particularly among individuals with high genetic risk, is an important area of scientific inquiry moving forward. Finally, 23andMe uses genotyping technology that comprises mostly European populations. This is a limitation in the field of genetics for genotyping and sequencing and has been discussed elsewhere (23andme.com/ancestry-composition-guide; 66).

## Conclusions

The current study presents an in-vivo approach to assessing participants’ attitudes and perceived utility about receiving personalized genetic information from a novel Genetics and Smoking Risk Profile (*RiskProfile*). Through detailed in-vivo, user-centered design feedback, we identified five inter-connected domains—the motivations, perceived utility, potential concerns, suggestions for improvement, and positive aspects of the *RiskProfile*. The *RiskProfile* was largely well received by participating current smokers and, with modifications that align with end-user feedback described herein, may hold promise as a useful smoking cessation tool in the future. This study highlights the advantages of using an in-vivo design to optimize the design of genetically-informed intervention tools and to maximize the rigor and relevance of end-user feedback when returning genetic information to participants.

## Supplementary Information

**Additional file 1.** **Supplemental Table 1.** Overview of the *RiskProfile*’s contents. **Supplemental Table 2.** Breakdown of initial codes, categories, and themes across questions. **Supplemental Text 1.** Details about coding with Microsoft Excel. **Supplementary Figure 1.** Reproduced with permission from Ramsey et al., 2020 (35).

## Data Availability

The datasets used and/or analyzed during the current study are available from the corresponding author on reasonable request.

## Abbreviations

COPD: Chronic Obstructive Pulmonary Disease
RiskProfile: Genetics and Smoking Risk Profile

## References

[1] Collins FS, Varmus H (2015). A new initiative on precision medicine. New England J Med.

[2] Peterson EB, Chou W-yS, Gaysynsky A, Krakow M, Elrick A, Khoury MJ, Kaphingst KA. Communication of cancer-related genetic and genomic information: A landscape analysis of reviews. 10.1093/tbm/ibx063 PMC6065548 29385592

[3] Jeibouei S, Akbari ME, Kalbasi A, Aref AR, Ajoudanian M, Rezvani A, Zali H (2019). Personalized medicine in breast cancer: pharmacogenomics approaches. Pharmacogen Per Med.

[4] Hamilton SP (2015). The promise of psychiatric pharmacogenomics. Bio Psych.

[5] Millican EA, Lenzini PA, Millian PE, Groxxo L, Eby C, Deych E (2007). Genetic-based dosing in orthopedic patients beginning warfarin therapy. Blood.

[6] Shahin MHA, Johnson JA (2013). Clopidogrel and warfarin pharmacogenetic tests: what is the evidence for use in clinical practice?. Current Opin Cardio.

[7] Hartz SM, Olfson E, Culverhouse R, Cavazos-Rehg P, Chen L-S, DuBois R (2015). Return of individual genetic results in a high-risk sample: Enthusiasm and positive behavioral change. Gen in Med.

[8] Moldovan R, Pintea S, Austin J (2017). The efficacy of genetic counseling for psychiatric disorders: a meta-analysis. J Gen Counsel.

[9] Norman P, Brain K (2005). An application of an extended health belief model to the prediction of breast self-examination among women with a family history of breast cancer. Brit J Health Psych.

[10] Chen L-S, Kaphingst KA, Tseng T-S, Zhao S (2016). How are lung cancer risk perceptions and cigarette smoking related? – testing an accuracy hypothesis. Transl Cancer Res.

[11] Roberts JS, Gornick MC, Carere DA, Wuhlmann WR, Ruffin MT, Green RC (2017). Direct-to-consumer genetic testing: user motivations, decision making, and perceived utility of results. Pub Health Genomics.

[12] Olfson E, Hartz S, Carere, DA, Green RC, Roberts JS, Bierut, LJ. Implications of personal genomic testing for health behaviors: the case of smoking. Nic Tobacco Res. 2016;2273–2277. 10.1093/ntr/ntw168 PMC5103936 27613923

[13] Khoury MJ, Iademarco MF, Riley WT (2016). Precision public health for the era of prediction medicine. Am J Prev Med.

[14] Belsky DW, Moffitt TE, Caspi A (2012). Genetics in population health science: Strategies and opportunities. Am J Pub Health.

[15] Meiser B, Guo XY, Putt S, Fulleton JM, Schofield PR, Mitchell PB, Yanes T. Psychosocial implications of living with familial risk of a psychiatric disorder and attitudes of psychiatric genetic testing: A systematic review of the literature. Am J Med Gen Part B. 2020;1–20. doi:10.1002/ajmg.b.32786 10.1002/ajmg.b.32786 32369270

[16] Waltz M, Meagher KM, Henderson GE, Goddard KAB, Muessig K, Berg JS, Weck KE, Cadigan RJ (2020). Assessing the implications of positive genomic screening results. Per Med.

[17] Stewart KFJ, Wesselius A, Schreurs MAC, Schols AMWJ, Zeegers MP (2018). Behavioral changes, sharing behavior and psychological responses after receiving direct-to-consumer genetic test results: a systematic review and meta-analysis. J Community Genet.

[18] O’Neill S, Lipkus IM, Sanderson SC, Shepperd J, Docherty S, McBride CM (2013). Motivations for genetic testing for lung cancer risk among young smokers. Tob Control.

[19] Kimball BC, Nowakowski KE, Maschke KJ, McCormick JB (2014). Genomic data in the electronic medical record: Perspectives from a biobank community advisory board. J Emp Res Hum Res Ethic.

[20] Prom-Wormley EC, Clifford J, Bourdon JL, Barr P, Blondino C, Ball J (2019). Developing community-based strategies with family health history: Assessing the association between community resident family history and interest in health education. Soc Sci Med.

[21] Salm M, Abbate K, Applebaum P, Ottmamn R, Chung W (2014). Use of genetic tests among neurologists and psychiatrists: Knowledge, attitudes, behaviors, and needs for training. J Gen Counsel.

[22] Zhou YZ, Wilde A, Meiser B, Mitchell PB, Barlow-Stewart K, Schofield PR (2014). Attitudes of medical genetics practitioners and psychiatrists toward communicating with patients about genetic risk for psychiatric disorders. Psych Gen.

[23] Fernandez CV, Strahlendorf C, Avard D, Knoppers BM, O’Connell C, Bouffet E (2013). Attitudes of Canadian researchers toward the return to participants of incidental and targeted genomic findings obtained in a pediatric research setting. Gen Med.

[24] Klitzman R, Appelbaum PS, Fyer A, Martinez J, Buquez B, Wynn J (2013). Researchers' views on return of incidental genomic research results: qualitative and quantitative findings. Gen Med.

[25] Nurnberger JI, Austin J, Berrettini WH, Besterman AD, DeLisi LE, Grice DE (2018). What should a psychiatrist know about genetics? Review and recommendations from the residency education committee of the International Society of Psychiatric Genetics. J Clin Psych.

[26] Wilson BJ, Islam R, Francis JJ, Grimshaw JM, Permaul JA, Allanson JE (2016). Supporting genetics in primary care: investigating how theory can inform professional education. Euro J Hum Gen.

[27] Kohler JN, Turbitt E, Lewis KL, Wilfond BS, Jamal L, Peay HL, Bieskecker LG, Biesecker BB (2017). Defining personal utility in genomics: a Delphi study. Clin Gen.

[28] Kaphingst KA, McBride CM, Wade C, Alford SH, Reid R, Lrason E, Baxevanis AD, Brody LC (2012). Patient understanding of and responses to multiplex genetic susceptibility test results. Genet Med.

[29] Kaphingst KA, McBride CM, Wade C, Alford SH, Brody LC, Baxevanis AD. Consumers’ use of web-based information and their decisiosn about multiplex genetic susceptibility testing. J Med Internet Res. 2010;12(3):e41. 10.2196/jmir.1587 PMC2956320 20884465

[30] Carere DA, Kraft P, Kaphinst KA, Roberts S, Green RC (2016). Consumers report lower confidence in their genetics knowledge following direct-to-consumer personal genomic testing. Genet Med.

[31] Frieser MJ, Wilson S, Vrieze SI (2018). Behavioral impact of return of genetic test results for complex disease: Systematic review and meta-analysis. Health Psychol.

[32] Visscher PM, Wray NR, Zhang Q, Sklar P, McCarthy MI, Brown MA, Yang J (2016). 10 years of GWAS discovery: biology, function, and translation. Am J Hum Gen.

[33] Finn CT, Smoller JW (2006). Genetic counseling in psychiatry. Harvard R Psych.

[34] Sperber NR, Carpetner JS, Cavallari LH, Damschroder LJ, Cooper-DeHoff RM, Denny JC, et al. Challenges and strategies for implementing genomic services in diverse settings: Experiences from the Implementing GeNomics in practice (IGNITE) network. BMC Med Gen. 2017;10(35). 10.1186/s12920-017-0273-2 PMC5441047 28532511

[35] International Society of Psychiatric Genetics [ISPG]. Genetic testing statement. 2017;Available from https://ispg.net/genetic-testing-statement/.

[36] Ramoni RB, McGuire AL, Robinson JO, Morley DS, Plon SE, Joffe S (2013). Experiences and attitudes of genome investigators regarding return of individual genetic test results. Gen Med.

[37] Sullivan PK, Daly MJ, O’Donovan M (2012). Genetic architectures of psychiatric disorders: the emerging picture and its implications. Nat R Gen.

[38] Kaphingst KA, Peterson E, Zhao J, Gaysynsky A, Elrick A, Hong SJ, Krakow M (2019). Cancer communication research in the area of genomics and precision medicine: a scoping review. Gen in Med.

[39] McBride CM, Graves KD, Kaphingst KA, Allen CG, Wang C, Arrendondo E, Klein WMP (2019). Behavioral and social scientists’ reflections on genomics: a systematic evaluation within the Society of Behavioral Medicine. RBM.

[40] Klein WMP, McBride CM, Allen CG, Arrendondo EM, Bloss CS, Kaphingst KA, Sturm AC, Wang C (2019). Optimal integration of behavioral medicine into clinical genetics and genomics. Am J Hum Gen.

[41] Senft N, Sanderson M, Selove R, Blot WJ, King S, Gilliam K (2019). Attitudes towards precision treatment of smoking in the Southern Community Cohort Study. Cancer Epi Biomark Prev.

[42] Waters EA, Ball L, Ghlert S. ‘I don’t believe it.’ Acceptance and skepticism of genetic health information among African-American and White smokers. Soc Sci Med. 2017;184:153–160. 10.1016/j.socscimed.2017.04.053 PMC5535773 28527373

[43] Shepperd JA, Novell CA, O’Neill SC, Docherty SL, Sanderson SC, McBride CM, Lipkus IM (2014). Contemplating genetic feedback regarding lung cancer susceptibility. Ann Behav Med.

[44] Bloss CS, Jeste DV, Schork NJ (2011). Genomics for disease treatment and prevention. Psych Clin North Am.

[45] Hollands GJ, French DP, Griffin SJ, Prevost AT, Sutton S, King S (2016). The impact of communicating genetic risks of disease on risk-reducing health behaviour: Systematic review with meta-analysis. BMJ.

[46] Lipkus IM, Schwartz-Bloom R, Kelley MJ, Pan W (2015). A Preliminary Exploration of College Smokers’ Reactions to Nicotine Dependence Genetic Susceptibility Feedback. Nic Tob Res.

[47] Sanderson SC, O’Neill SC, White DB, Depler G, Bastian L, Lipkus IM, McBride CM (2009). Responses to online GSTM1 genetic test results among smokers related to patients with lung cancer: a pilot study. Cancer Epi Biomarkers Prev.

[48] Kong C, Dunn M, Parker M (2017). Psychiatric genomics and mental health treatment: setting the ethical agenda. Am J Bioethics.

[49] Roberts MC, Kennedy AE, Chambers DA, Khoury MJ (2017). The current state of implementation science in genomic medicine: opportunities for improvement. Gen in Med.

[50] Austin JC. Evidence-based genetic counseling for psychiatric disorders: A road map. Cold Spring Harbor P Med. 2019;1–14. 10.1101/cshperspect.a036608 PMC7263094 31501264

[51] Ramsey AT, Chen L-S, Hartz SM, Saccone NL, Fisher SL, Proctor EK, Bierut LJ (2018). Toward the implementation of genomic applications for smoking cessation and smoking-related diseases. TBM.

[52] Ramsey AT, Bourdon JL, Bray M, Dorsey A, Zalik M, Pietka A (2021). Proof of concept of a personalized genetic risk tool to promote smoking cessation: High acceptability and reduced cigarette smoking. Cancer Prev Res.

[53] Ramsey AT, Bray M, Laker PA, Bourdon JL, Dorsey A, Zalik M (2020). Participatory design of a personalized genetic risk tool to promote behavioral health. Cancer Prev Res.

[54] Strauss AL, Corbin JM (1998). Basics of qualitative research: techniques and procedures for developing grounded theory.

[55] Hsieh H-F, Shannon SE (2005). Three approaches to qualitative content analysis. Qual Res.

[56] Myer DZ, Avery LM (2009). Excel as a qualitative data analysis tool. Field Methods.

[57] Bree R, Gallagher G (2016). Using Microsoft Excel to code and thematically analyze qualitative data: a simple, cost-effective approach. AISHE-J.

[58] Moller AC, Merchant G, Conroy DE, West R, Hekler E, Kugler KC (2017). Applying and advancing behavior change theories and techniques in the context of a digital health revolution: Proposals for more effectively realizing untapped potential. J Behav Med.

[59] Zhang C-Q, Zhang R, Schwarzer R Hagger MS. A meta-analysis of the health action process approach. Health Psychol. Off. J. Div. Health Psych Am Psych Assoc. 2019;38:623–637. 10.1037/hea0000728 30973747

[60] Chiu A, Hartz S, Smock N, Chen J, Qazi A, Onyeador J (2018). Most current smokers desire genetic susceptibility testing and genetically-efficacious medication. J Neuroimmune Pharm.

[61] Michie S, Van Stralen MM, West R (2011). The behaviour change wheel: a new method for characterising and designing behaviour change interventions. Impl Sci.

[62] Rosenstock IM, Strecher VJ, Becker MH (1988). Social learning theory and the health belief model. Health Edu Quarterly.

[63] Marteau TM, French DP, Griffin SJ, Prevost AT, Sutton S, Watkinson C, Attwood S, Hollands GJ (2010). Effects of communicating DNA-based disease risk estimates on risk-reducing behaviors. Cochrane.

[64] Cornetta K, Brown CG (2013). Balancing personalized medicine and personalized care. Acad Med.

[65] Costain G, Esplen MJ, Toner B, Hodgkinson KA, Bassett AS (2014). Evaluating genetic counseling for family members of Individuals with Schizophrenia in the molecular age. Schizophrenia Bull.

[66] Peterson RE, Kuchenbaecker K, Walters RK, et al. Genome-wide association studies in ancestrally diverse populations: Opportunities, methods, pitfalls, and recommendations. Cell. 2019;179. 10.1016/j.cell.2019.08.051 PMC6939869 31607513

