# Supplementary material for: In-vivo design feedback and perceived utility of a genetically-informed smoking risk tool among current smokers in the community
Source: BMC Med Genomics. 2021 May 26;14:139. doi: 10.1186/s12920-021-00976-1 (PMC8152342; doi:10.1186/s12920-021-00976-1)

**Supplement**

| *Supplemental Table 1.* Overview of the *RiskProfile*’s contents. | | |
| --- | --- | --- |
| Location | Section | Details |
| Outer panel | Introduction | Primed participants for what to expect in the tool (information about their genetics and smoking-related outcomes). |
| Inner flap | Overview of chromosomes | Listed all 23 human chromosomes with chromosome 15 highlighted because it was the location of the variants of interest for this study. |
| Inner panel (across all three) | Participants’ personalized risk score | There was one score each for: lung cancer, lung disease (chronic obstructive pulmonary disease), and difficulty quitting smoking. Scores ranged from green (lowest risk) to red (highest risk). The risk score was based on an algorithm that took both genetic (from 23andMe) and environmental (number of cigarettes per day at visit 1) information into account. |
| Inner panel (left) | Participants’ personalized genetic information | Based on the 23andMe raw data (see below), we assessed varying genetic markers for participants of European ancestry (rs16969968, rs75106522, rs11631955), African ancestry (rs16969968 and rs2036527), and any other (non-European/non-African) ancestry (rs16969968). This section included each participants’ results for the ancestry-dependent genetic markers examined in their 23andMe results (e.g., rs16969968), chromosomal location of each marker (e.g., Chromosome 15), possible genetic variants for each marker (e.g., G or A), the risk variant for each marker (e.g., A), and the participant’s actual genetics for each marker (e.g., G/A). |
| Inner panel (across middle and right) | Benefits of quitting smoking | Emphasized the importance of cessation regardless of a participant’s genetic makeup as well as the duration, frequency, and heaviness of their smoking. |
| Back panel | Resources to help with smoking cessation | Included toll-free numbers, apps, and possible medications for participants to bring up at their next doctor’s appointment. |

| *Supplemental Table 2.* Breakdown of initial codes, categories, and themes across questions. | | | | |
| --- | --- | --- | --- | --- |
| Question | Number of Codes | Number of Categories | Number of Themes | Relevant Domains |
| Question 1: When you first signed up for this study, what did you most want to learn in regard to your smoking behaviors? | 22 codes | 5 categories | 4 themes | Domains 1, 5 |
| Question 2: How might a tool like this genetics and smoking risk profile be helpful in in guiding smoking cessation attempts? | 33 codes | 4 categories | 3 themes | Domains 2, 4, 5 |
| Question 3: What are some of your concerns, if you have any, on using a tool like this profile to guide smoking cessation attempts? | 31 codes | 8 categories | 4 themes | Domains 3, 4, 5 |
| Question 4: How could this genetics and smoking risk profile be improved to help motivate smoking cessation attempts? | 34 codes | 9 categories | 3 themes | Domains 4, 5 |

*Supplemental Text 1.* Details about coding with Microsoft Excel.

One Excel document had one per question with participants’ IDs and responses copied into the first few columns of each sheet. Three blank columns were inserted to the right of the responses to track codes, and more columns could be inserted as necessary. Coders filled in at least once code per response in the first column (e.g., “motivator to quit,” “look at more markers,” “none”). To the right of the “raw code” columns was a column that listed each code along with the number of responses that used that code (e.g., “positive feedback = 5”). This allowed coders to quickly view the list of codes when reviewing the data. To the right of that, categories were listed in a column along with the codes that fell under that category (e.g., “Can be used to motivate = motivator to quit, find ways to quit, general motivator, change lifestyle”). Themes were listed in the final column.

*Supplementary Figure 1*. Reproduced with permission from Ramsey et al., 2020 (35).


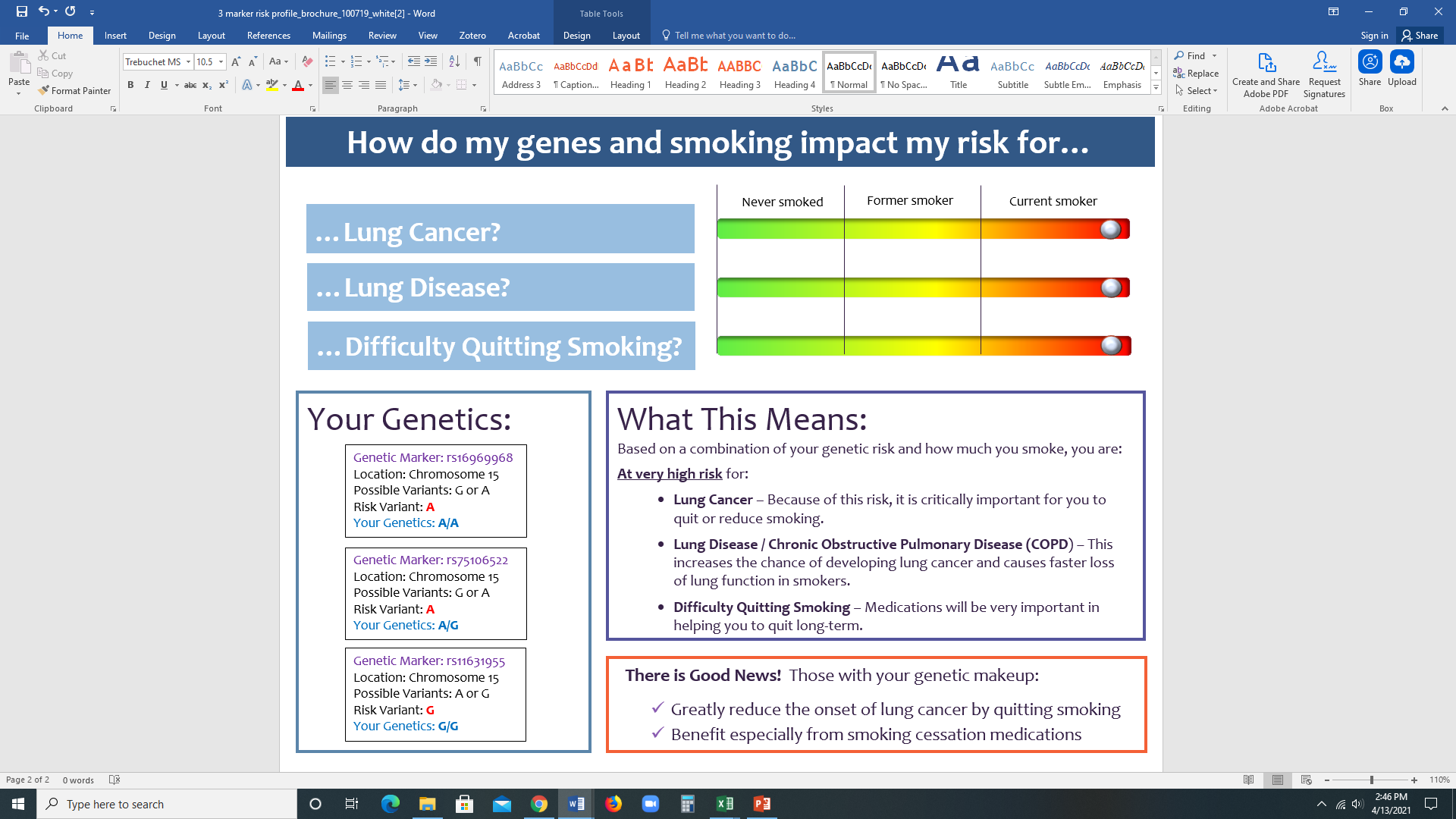

Supplement: Supplementary file 1 — Additional file 1. Supplemental Table 1. Overview of the RiskProfile’s contents. Supplemental Table 2. Breakdown of initial codes, categories, and themes across questions. Supplemental Text 1. Details about coding with Microsoft Excel. Supplementary Figure 1. Reproduced with permission from Ramsey et al., 2020 (35). [file 12920_2021_976_MOESM1_ESM.docx]
